# Bibliometric analysis of research hotspots and global trends in patient safety education for nursing students

**DOI:** 10.1097/MD.0000000000040163

**Published:** 2024-10-18

**Authors:** Ying Wang, Yi-Wen Chen, Xin-Ping Hu, Hua Mei

**Affiliations:** a Shanghai University of Traditional Chinese Medicine, 201203, Shanghai, China; b Shanghai University of Medicine and Health Sciences, Shanghai, China.

**Keywords:** bibliometrics, education, nursing students, patient safety

## Abstract

Patient safety education for nursing students has recently garnered interest. However, there is a paucity of data on the key areas and global trends over time. This study aims to analyze research patterns in the field of patient safety education for nursing students, identifying key contributors and global trends. A descriptive bibliometric approach was employed to analyze 782 articles related to patient safety education using data from the Web of Science Core Collection and CiteSpace 6.2. R4. Co-occurrence and co-citation analyses were conducted to identify prominent authors, institutions, countries, and common keywords. The analysis revealed contributions from 335 authors and 302 institutions across 78 countries. Levett-Jones T emerged as the most prolific author with 12 articles. The United States and the University System of Ohio were identified as the most productive countries and institutions, with 276 and 26 articles, respectively. Frequently occurring keywords included patient safety, education, medical education, simulation training, and standardized patients. This study provides valuable insights into the global research landscape of patient safety education in nursing, highlighting key contributors and trends. These findings can assist nursing students, educators, and researchers in identifying potential collaborators and informing future research to advance patient safety education in nursing.

## 1. Introduction

Patient safety represents a paramount concern in the field of healthcare. In May 2021, the 74th World Health Assembly adopted the Global Action Plan for Patient Safety 2021-2030, which aims to strengthen patient safety as a critical element of global health system design, processes and performance assessment.^[1]^ This strategic plan provides guidance to governments, health sector entities, World Health Organization (WHO) and the WHO Secretariat on the implementation of the resolution on patient safety. The plan aims to improve the quality and safety of global health systems, including diagnosis, treatment and care. This plan of action illustrates the crucial importance of reducing the incidence of preventable harm and ensuring patient safety for healthcare organizations.^[2–4]^ It is evident that a significant proportion of previously reported adverse medical events and associated economic losses, deaths and injuries globally could have been prevented. In accordance with the WHO, patient safety is defined as the absence of preventable harm to a patient during the course of healthcare and the reduction of the risk of unnecessary harm associated with healthcare to an acceptable minimum.^[5]^ International guidelines on patient safety highlight the necessity for all healthcare professionals to possess a fundamental understanding of patient safety practices.

The impact of nurses on patient safety can be directly observed in healthcare facilities, as nurses represent the largest workforce in these facilities and are the population most closely associated with patients. Therefore, it can be argued that nurses are critical to ensuring patient safety in healthcare facilities.^[6]^ Despite the rapid advances in medical technology that continue to improve patient outcomes, these advances are accompanied by complex risk interactions. This has led to the implementation of patient safety education in the United States, the United Kingdom, Canada, New Zealand, Australia, and Japan.^[2,3,7,8]^ This education is provided to undergraduate and graduate students at all levels. While the content and approach may differ from country to country, the objective is to develop patient safety awareness and related skills. The global social crisis precipitated by the emergence of the COVID-19 pandemic in 2020 has had a profound impact on human health and healthcare systems.^[9]^ The necessity for healthcare workers to undergo comprehensive training during periods of high patient influx has posed a challenge to the maintenance of patient safety, resulting in an increase in workload and medical errors.

To meet the needs of 21st century patients, the modern healthcare paradigm has shifted to a patient-centered care model that emphasizes interdisciplinary teams, evidence-based practices, and improved safety and quality. Medical students form the foundation of healthcare, while nursing students are focused on providing medical care. Globally, it is imperative that nursing students receive comprehensive patient safety education during their academic training. This education is critical to minimizing adverse patient events, reducing physical and psychological harm, and promoting recovery.

Bibliometrics is increasingly being applied across disciplines and is particularly effective in the mapping of scientific fields. This approach helps to manage the vast, fragmented, and sometimes controversial streams of research, given the current focus on empirical contributions. Bibliometric metrics have become essential for assessing scholarly productivity in today’s academic landscape. These parameters are measures of the output of individual authors and of the quality and impact of their publications. In addition, bibliometrics help researchers understand the prevailing trends and dynamics within their research fields. This allows them to propose more effective research topics.

Despite its potential, a bibliometric analysis for the identification of hotspots and global trends in nursing student education in the area of patient safety has not yet been conducted. It is expected that potential research partners, institutions and countries for collaborative research will be identified from the results of this study. This will be of benefit to nursing students, medical educators, researchers and academics. In addition, the data will enhance the understanding of the current literature on patient safety education in nursing. This will inform future research and contribute to the advancement of patient safety education.

## 2. Materials and methods

### 2.1. Description of outcome

The aim of this study was to identify hotspots and global trends in patient safety education for nursing students and to provide references on this topic for researchers, academics, and practitioners. The authors, cited authors, countries, institutions, cited journals, cited references, and keywords of the included articles are summarized and visualized. The following questions have been addressed in this research project.

### 2.2. Design

This study is a descriptive bibliometric research study. The data for this study were obtained from the Web of Science (WoS) Core Collection database, which is widely recognized as a leading global research indexing database, so this study involved no participating individuals. The WoS is renowned as the largest and most established repository of research publications and citations worldwide, commonly utilized in bibliometric analyses. This comprehensive database encompasses a curated selection of scholarly journals, reviews, conference proceedings, and books. Additionally, the WoS includes prominent journal citation indexes such as the Social Sciences Citation Index, Science Citation Index Expanded, Arts and Humanities Citation Index, and Emerging Sources Citation Index.^[10]^ As a result, data extraction was carried out from the WoS.

### 2.3. Search strategy

The data search was performed on May 1, 2024. In the search strategy, we used keywords based on MeSH terms. The data search strategy was: TS=[“patient safety education” AND “nursing student” OR “nurse students” OR “nursing students”] inclusion criteria were as follows: articles on patient safety health education for nursing students; the document type included articles; the article’s language was set to English; the WoS category was selected as “Nursing” category; and the time span of the articles was limited to 2013 to 2023. Exclusion criteria were as follows: early access, proceeding papers, book, book chapters, review articles, and editorial materials; and duplicate articles.

### 2.4. Data collection

Data collection was conducted by 2 authors, both postgraduate nursing students with academic research experience in systematic review and meta-analysis. included and excluded articles were independently reviewed by 2 authors, and any disagreements between the authors were handled by a third author until an agreement was reached. First, the studies were independently reviewed by titles or abstracts in the WoS database. After that, the studies in the WoS were extracted as plain text files with full records and cited references from the WoS. Duplicated articles were detected and removed with CiteSpace 6.2.R4 software.^[11]^ Each item contained authors names, article titles, journal titles, abstracts, keywords, references, organization, funding, and citation information.

### 2.5. Data analysis

The data analysis was conducted using CiteSpace software version 6.2.R4.^[12]^ The parameters used in CiteSpace included setting the time slicing from January 2013 to December 2023, with 1 year per slice and selecting the top 50 items for yearly citation rank. Co-citation analysis and co-occurrence network were performed on node types such as authors, cited authors, countries, institutions, cited journals, cited references, and keywords. The co-citation analysis aimed to identify the basis of the research area and determine the most relevant documents to the cited literature. A co-occurrence network analysis was carried out to identify leading authors, countries, and institutions. Cluster analysis was also conducted to understand hotspots and research developments in this field of study. Additionally, citation burst analysis was performed to identify the most active articles and areas within this research area.

The quality and statistical significance of the network structure and performance are calculated by the network density, modularity Q, and mean silhouette values.^[13]^ The modularity Q value is from 0 to 1. If the value is >0.3, it is an indication that the cluster structure is significant. The mean silhouette value is in the range from −1 to +1. A value >0.7 indicates that the cluster is strong and credible.^[14]^ Additionally, betweenness centrality is a parameter used to determine influential nodes and measure a node’s centrality on a path connecting other nodes in the network. In CiteSpace software, larger nodes indicate greater frequency, thick purple circles indicate greater betweenness centrality and thick red circles indicate greater citation bursts. Stronger collaborations or co-citations are indicated by a thicker line.

### 2.6. Validity and reliability

All analyses in the study were based on quantitative data. Two independent authors participated in the data collection and analysis, and any disagreements between the authors were resolved by a third author until agreement was reached. As a result, the results were reliable and credible.

### 2.7. Ethical considerations

The bibliometric research method was not subject to the requirement of ethics approval.

## 3. Results

**Figure 1.**
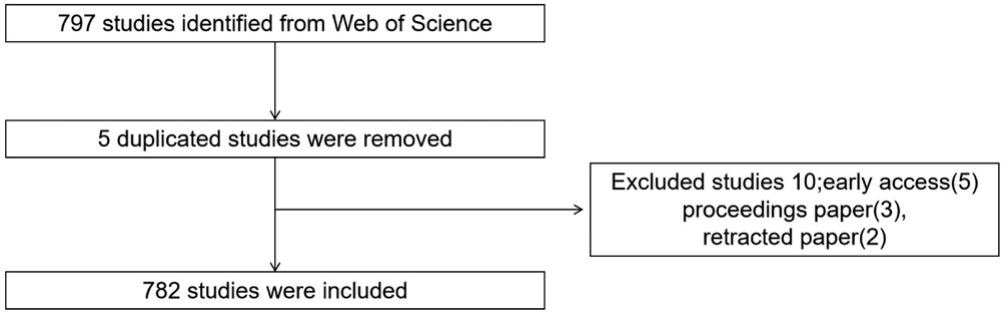
Flow diagram of study selection.

**Figure 2.**
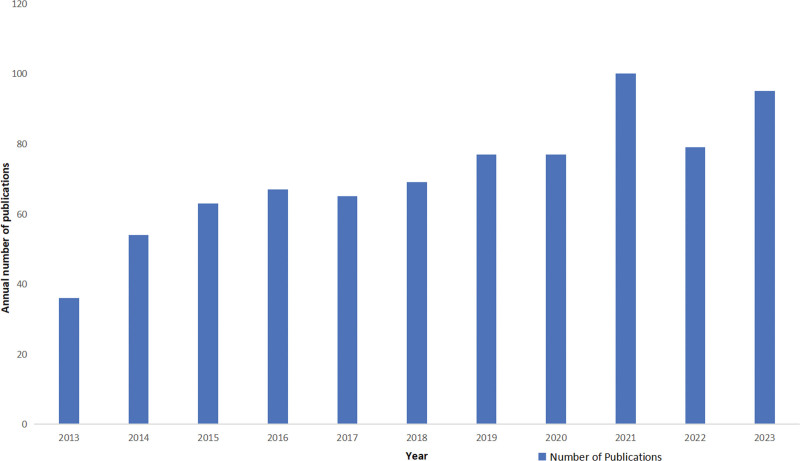
Annual distribution of a total of 782 articles on patient safety education for nursing students from 2013 to 2023.

The flowchart used to select the studies is shown in Figure 1. A total of 797 articles were identified and no duplicate articles were found. A total of 15 articles were then excluded for the following reasons: Five were duplicates, 5 were early access articles, 3 were proceedings articles, and 2 were retracted articles. The bibliometric analysis included 782 articles. The annual distribution of patient safety and nursing student health education articles from 2013-2023 is shown in Figure 2. The number of publications in this area showed a gradual increase between 2013 and 2023. The number of publications peaked in 2021 with 100 articles. This indicates that patient safety and health education are topics of significant interest to nursing students.

### 3.1. Distribution by authors and co-cited authors

**Figure 3.**
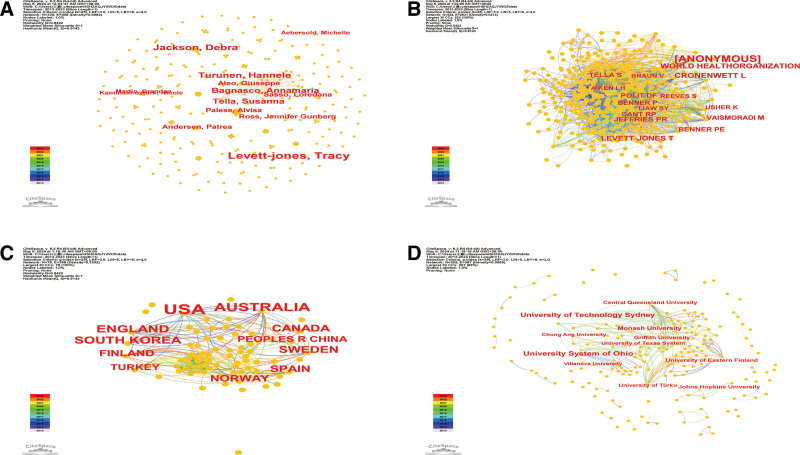
(A) The network map of the authors on patient safety education for nursing students from 2013 to 2023. (B) The network map of the co-citation authors on patient safety education for nursing students from 2013 to 2023. (C) The network map of the countries on patient safety education for nursing students from 2013 to 2023. (D) The network map of the institutions on patient safety education for nursing students from 2013 to 2023.

The 782 articles on patient safety education for nursing students were performed by 335 authors. The network map of the authors and co-citation authors is presented in Figure 3A and Figure 3B, the top 10 authors and co-citation authors are listed in Table 1. The most productive author was Levett-Jones, Tracy with 12 articles. The second productive author was Jackson, Debra with 8 articles. The third productive author was Turunen, Hannele with 7 articles.

**Table 1:** Top 10 authors, co-cited authors, countries, and institutions on patient safety education for nursing students.

Rank	Author				Co-cited author					
	Sort by no. of articles published		Frequency		Sort by no. of cited articles		Citation	Sort by centrality		Centrality
1	Levett-Jones, Tracy		12		Cronenwett L		83	Cant Rp		0.11
2	Jackson, Debra		8		World Health Organization		80	Bandura A		0.11
3	Turunen, Hannele		7		Levett-Jones T		66	Benner P		0.09
4	Bagnasco, Annamaria		6		Jeffries Pr		62	Cronenwett L		0.08
5	Tella, Susanna		6		Benner Pe		51	Levett-Jones T		0.08
6	Palese, Alvisa		4		Tella S		49	Aiken Lh		0.08
7	Andersen, Patrea		4		Cant Rp		49	Benner Pe		0.07
8	Aleo, Giuseppe		4		Polit Df		48	Vaismoradi M		0.07
9	Ross, Jennifer Gunberg		4		Benner P		44	Agency For Healthcare Research And Quality		0.07
10	Sasso, Loredana		4		Vaismoradi M		40	Cook Da		0.07
	Countries						Institutions			
Rank	Sort by no. of articles published	Frequency		Sort by centrality		Centrality	Sort by no. of articles published	Frequency	Sort by centrality	Centrality
1	United States of America	276		United States of America		0.2	University System of Ohio	26	University of Texas System	0.17
2	Australia	106		Australia		0.2	University of Technology Sydney	21	University System of Ohio	0.11
3	England	64		Turkey		0.19	Monash University	13	University of Technology Sydney	0.11
4	South Korea	53		Italy		0.16	Griffith University	10	University of Turku	0.10
5	Canada	43		Saudi Arabia		0.13	Johns Hopkins University	10	Flinders University South Australia	0.07
6	Sweden	42		Sweden		0.12	University of Eastern Finland	10	Central Queensland University	0.06
7	Spain	38		England		0.11	University of Turku	9	Karolinska Institute	0.04
8	Norway	36		Norway		0.11	University of Texas System	9	University of Massachusetts System	0.04
9	Finland	31		Spain		0.09	Central Queensland University	9	American University of Beirut	0.04
10	Turkey	26		Thailand		0.09	Villanova University	8	Monash University	0.03

**Figure 4.**
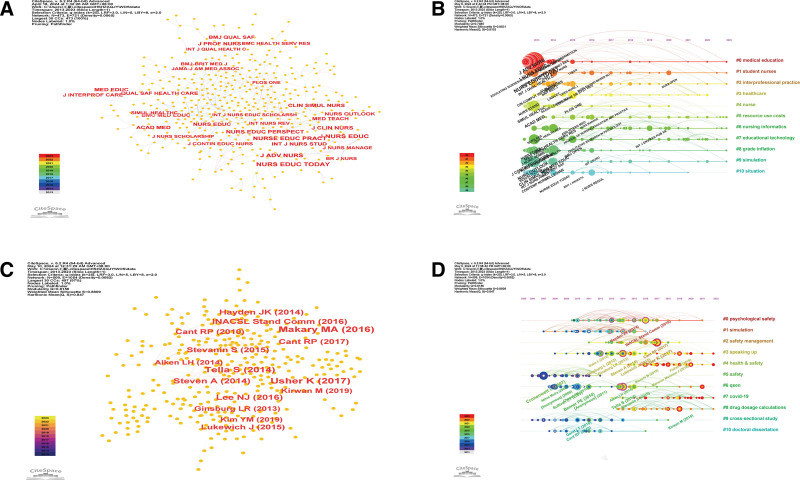
(A) The network map of the journal on patient safety education for nursing students from 2013 to 2023. (B) The chronological order in which the journals on patient safety education for nursing students from 2013 to 2023 appear in each cluster. (C) The network map of the cited journals on patient safety education for nursing students from 2013 to 2023. (D) The chronological order in which the cited journals on patient safety education for nursing students from 2013 to 2023 appear in each cluster.

The top 5 authors with the largest number of citations were Cronenwett L (83 citations), WHO (80 citations), Levett-Jones T (66 citations), and Jeffries Pr (62 citations). The top 3 co-cited authors in terms of centrality were Cant Rp (0.11), Bandura A (0.11), and Benner P (0.11) (Table 1). The 3 authors who had the strongest citation burst were Levett-Jones, Tracy (strength 4.28, 2018–2019), Turunen, Hannele (strength 2.46, 2014–2015), and Jackson, Debra (strength 2.35, 2017–2020) (Fig. 4).

### 3.2. Analysis of countries and institutions

The 782 articles on patient safety education for nursing students were performed at 302 institutions in 78 countries. The network map of the countries and institutions is shown in Figure 3C and Figure 3D and the top 10 are listed in Table 1. The United States of America (USA) with 276 articles had the largest number of articles published, followed by Australia with 106 articles and England with 64 articles. The top 3 countries in terms of centrality were the USA and Australia (each with 0.2), and Turkey (0.19) (Table 1). The country with the highest citation burst was Spain (strength 5.38, 2021–2023) (Fig. 4).

The top 3 institutions with the highest number of publications were the University System of Ohio with 26 articles, University of Technology Sydney with 21 articles, and C Monash University with 13 articles. The top 3 institutions in terms of centrality were the University of Texas System (0.17), the University System of Ohio, and the University of Technology Sydney (each with 0.11) (Table 1). The University of Technology Sydney (strength 5.77, 2018–2019), Eastern Finland (strength 3.88, 2014–2016), the University of Turku (strength 2.97, 2021–2023), and the University System of Ohio (strength 1.96, 2013–2016) had the strongest citation bursts (Fig. 4).

### 3.3. Analysis of journals and cited journals

The journals with the most published articles and the network map of the cited journals are shown in Figure 4A and the top 10 are listed in Table 2. The top 3 journals publishing patient safety education for nursing students are *Nurse Education Today* with 125 articles, the *Nurse Education in Practice* with 66 articles, and *Clinical Simulation in Nursing* with 57 articles. The top 3 cited journals were the *Nurse Education Today* (520 citations), the *Journal of Nursing Education* (387 citations), and the *Journal of Advanced Nursing* (336 citations). The *British Medical Journal* (0.08) had the highest centrality. The chronological order in which the cited journals appear in each cluster are shown in Figure 4B. The *International Journal of Environmental Research and Public Health* (strength 11.46,2021–2023), the *Nursing Open* (strength 8.22, 2021–2023), and the *Clinical Simulation in Nursing*(strength 6.14, 2013–2017) had the strongest citation bursts (Fig. 4).

**Table 2:** Top 10 journals and cited journals on patient safety education for nursing students.

	Journal		Cited Journal			
Rank	Sort by no. of articles published	Frequency	Sort by no. of cited articles	Citation	Sort by centrality	Centrality
1	*Nurse Education Today*	125	*Nurse Education Today*	520	*British Medical Journal*	0.08
2	*Nurse Education In Practice*	66	*Journal Of Nursing Education*	387	*Annals of Internal Medicine*	0.07
3	*Clinical Simulation In Nursing*	57	*Journal Of Advanced Nursing*	336	*Nurse Educator*	0.06
4	*Journal Of Nursing Education*	50	*Nurse Education In Practice*	322	*American Journal Of Critical Care*	0.05
5	*Nurse Educator*	34	*journal of clinical nursing*	237	*Clinical Simulation in Nursing*	0.05
6	*Journal Of Clinical Nursing*	32	*Nursing Education Perspectives*	213	*BMC Health Services Research*	0.05
7	*Journal Of Interprofessional Care*	27	*International Journal of Nursing Studies*	200	*American Journal of Nursing*	0.05
8	*BMC Medical Education*	25	*Journal Of Professional Nursing*	190	*Computers Informatics Nursing*	0.05
9	*Journal Of Professional Nursing*	23	*Clinical Simulation in Nursing*	188	*Joint Commission Journal on Quality and Patient Safety*	0.05
10	*BMC Nursing*	17	*Academic Medicine*	176	*Cochrane Database of Systematic Reviews*	0.05

### 3.4. Analysis of cited references

The network map of the cited references is presented in Figure 4C and the top 10 are listed in Table 3. The 3 most commonly cited references on this topic were Cronenwett and Sherwood (2016) with 34 citations, Makary and Daniel (2016) with 30 citations, and Usher and Woods (2017) with 29 citations. Aiken and Sloane (2014; 0.26), Tella and Liukka (2014; 0.21), and Steven and Magnusson (2014; 0.18) had high centrality in this research area (Table 3). The top 3 references with the strongest citation bursts were in psychological safety, simulation, and safety management (Fig. 4D).

**Table 3:** Top 10 Co-Cited References on patient safety education for nursing students

Rank	Author	Year	Reference	Citation	Centrality
Sort by no. of citation					
1	Cronenwett and Sherwood	2007	Quality and safety education for nurses	34	0.01
2	Makary and Daniel	2016	Medical error-the third leading cause of death in the US	30	0.02
3	Usher and Woods	2017	Self-reported confidence in patient safety knowledge among Australian undergraduate nursing students: A multisite cross-sectional survey study	29	0.11
4	Benner	2010	Educating Nurses: A Call for Radical Transformation – How Far Have We Come?	28	0.01
5	Tella and Liukka	2014	What do nursing students learn about patient safety? an integrative literature review	25	0.21
6	Steven and Magnusson	2014	Patient safety in nursing education: contexts, tensions and feeling safe to learn	21	0.18
7	Lee and Jang	2016	Patient safety education and baccalaureate nursing students’ patient safety competency: A cross-sectional study	21	0.11
8	INACSL Standards Committee	2016	INACSL Standards of Best Practice: SimulationSM Simulation Design	20	0.17
9	Cant and Cooper	2017	Use of simulation-based learning in undergraduate nurse education: An umbrella systematic review	18	0.02
10	Stevanin and Bressan	2015	Knowledge and competence with patient safety as perceived by nursing students: The findings of a cross-sectional study	18	0.04
Sort by no. of centrality					
1	Aiken and Sloane	2014	Nurse staffing and education and hospital mortality in 9 European countries: a retrospective observational study	14	0.26
2	Tella and Liukka	2014	What do nursing students learn about patient safety? an integrative literature review	25	0.21
3	Steven and Magnusson	2014	Patient safety in nursing education: contexts, tensions and feeling safe to learn	21	0.18
4	INACSL Standards Committee	2016	INACSL Standards of Best Practice: SimulationSM Simulation Design	20	0.17
5	Bogossian and Cooper	2014	Undergraduate nursing students’ performance in recognizing and responding to sudden patient deterioration in high psychological fidelity simulated environments: An Australian multi-center study	6	0.14
6	Foronda and Liu	2013	Evaluation of Simulation in Undergraduate Nurse Education: An Integrative Review	7	0.13
7	Fey and Gloe	2015	Assessing the Quality of Simulation-Based Research Articles: A Rating Rubric	4	0.12
8	James	2013	A New, Evidence-based Estimate of Patient Harms Associated with Hospital Care	99	0.12
9	Lee and Jang	2016	Patient safety education and baccalaureate nursing students’ patient safety competency: A cross-sectional study	21	0.11
10	Usher and Woods	2017	Self-reported confidence in patient safety knowledge among Australian undergraduate nursing students: A multisite cross-sectional survey study	29	0.11

### 3.5. Analysis of keywords

**Figure 5.**
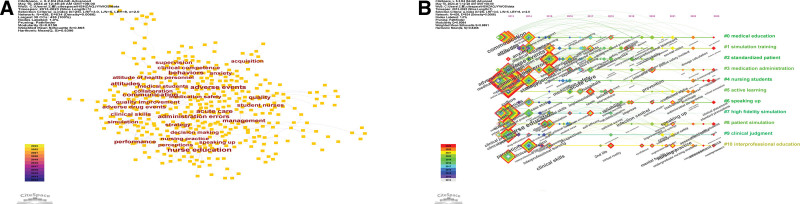
(A) The network map of keywords engaged in patient safety education for nursing students. (B) The chronological order in which the keywords on patient safety education for nursing students from 2013 to 2023 appear in each cluster.

**Figure 6.**
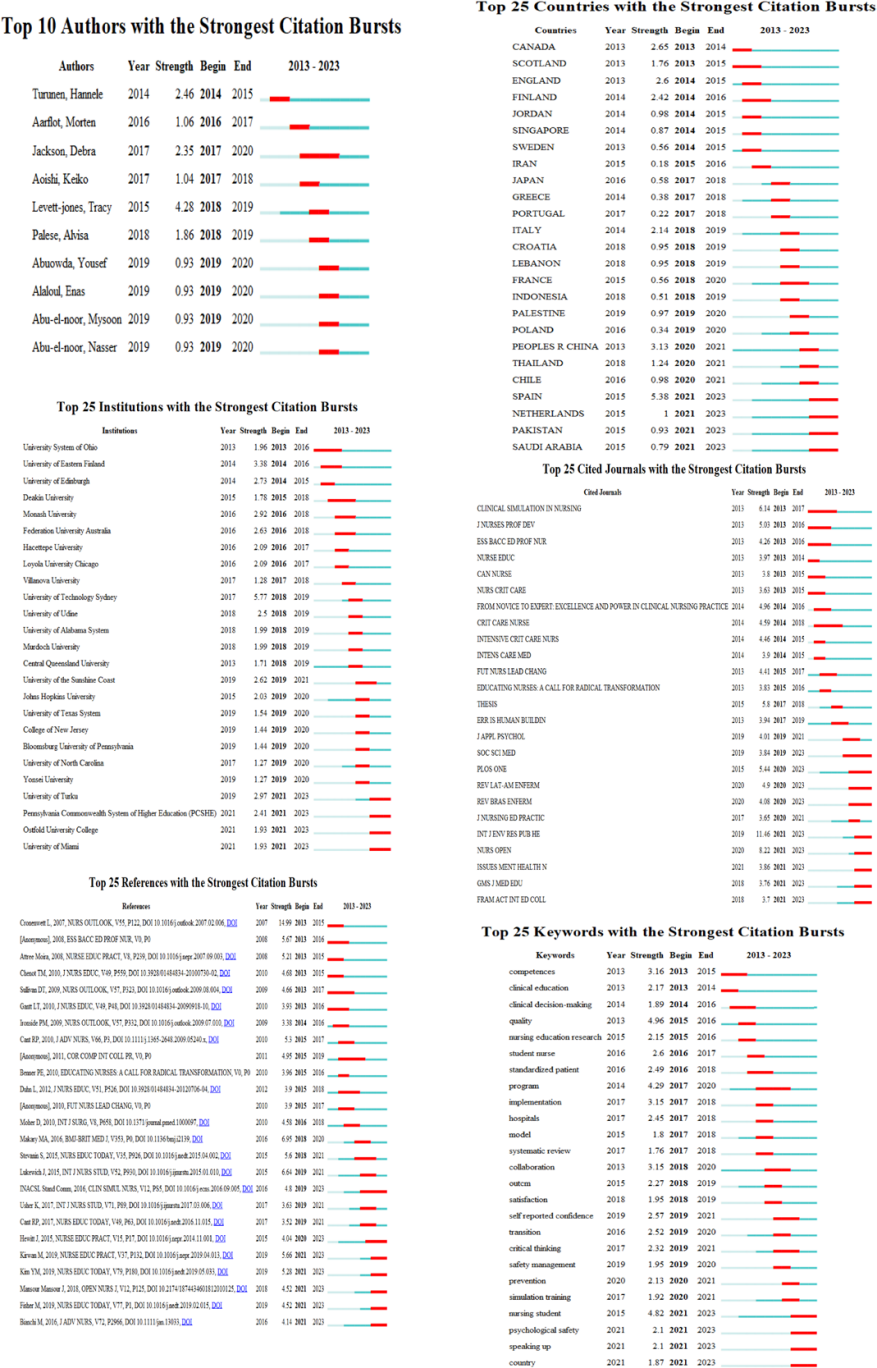
The strongest citation bursts on patient safety education for nursing students from 2013 to 2023.

The network map of the keywords is shown in Figure 5A and the top 20 keywords are listed in Table 4. The top 3 keywords were patient safety with 300 articles, education with 247 articles, and nursing education with 182 articles. The top 3 keywords in terms of centrality were adverse events (0.43), acute care (0.36), and medical students (0.21) (Table 4). The keywords of quality (strength 4.96, 2015–2016), nursing student (strength 4.82, 2021–2023), and competence (strength 3.16, 2013–2015) had the strongest citation bursts (Fig. 6).

**Table 4:** Top 20 keywords on patient safety education for nursing students by frequency and centrality.

Rank	Keywords	Frequency	Keywords	Centrality
1	Patient safety	300	Adverse events	0.43
2	Education	247	Acute care	0.36
3	Nursing education	182	Medical students	0.21
4	Nursing students	156	Clinical decision making	0.2
5	Nurses	130	Behaviors	0.19
6	Students	122	Experience	0.18
7	Care	107	Clinical education	0.17
8	Knowledge	76	Baccalaureate	0.17
9	Perceptions	75	Communication	0.16
10	Simulation	71	Nurse education	0.14
11	Quality	66	Management	0.14
12	Safety	63	Medication safety	0.14
13	Experiences	61	4th year	0.14
14	Competence	59	Medical errors	0.13
15	Interprofessional education	56	Decision making	0.13
16	Skills	55	Blood transfusion	0.13
17	Errors	54	Clinical skills	0.12
18	Health care	50	Supervision	0.12
19	Impact	47	Attitude of health personnel	0.12
20	Attitudes	46	Quality	0.11

A total of 20 clusters were obtained from the cluster analysis. The most common theme in keywords on patient safety education for nursing students was medical education with 33 articles. The top 3 keywords in this cluster were health care, and attitude and communication. The second most common theme in this research area was simulation training with 13 articles, and medication error and nursing student and adverse events were the top keywords in this theme. The third theme was standardized patient with 6 articles, knowledge and experience, and critical thinking (Fig. 5B).

## 4. Discussion

We performed a bibliometric analysis of 782 articles on patient safety education for nursing students published between 2013 and 2023 using the WoS databases and CiteSpace. To the best of our knowledge, this study is the first to examine the bibliometric parameters, citation trends, scientific output, and development trends of patient safety education for nursing students. Thus, this study can help in identifying the most prolific authors, institutions, countries, new trends in patient safety education for nursing students, patterns of collaboration, and journals publishing patient safety education for nursing students for researchers, practitioners, and scholars. In addition, this study provides important insights into gaps in the field and helps to identify and define the main themes of this research area.

The analysis showed that research in patient safety education for nursing students has steadily increased between 2013 and 2023, with a significant spike in 2021, producing 100 articles. Furthermore, recognizing patient safety accurately is achieved by detecting errors and gathering necessary information, which can be directly related to communication for patient safety. Confidence and opinions about patient safety cannot be established in a short time.^[15]^ Moreover, as nursing education has evolved, it is evident that the scope of patient safety education for nursing students has evolved in parallel with modern healthcare demands, particularly in light of increasing life expectancy, multimorbidity, aging populations, and the postpandemic era.^[16]^

### 4.1. Collaboration and network analysis

Author cooperation network analysis revealed that 335 authors were involved in research on patient safety education for nursing students, yet no strong collaborations existed between them. Advancing nursing research can be expedited through enhanced collaboration among institutional units within a country. Establishing international research collaborations fosters cross-cultural learning and strengthens global research efforts, addressing emerging challenges in healthcare.^[17]^ Greater international cooperation can broaden the scope of research, stimulate pertinent inquiries, and jointly develop solutions for international nursing issues, which would be difficult to achieve individually. Moreover, key contributors to the field, such as Cant, Roper, Bandura, and Benner, were identified. Their work provides critical insights into the structure of the field, and future researchers are encouraged to explore their contributions.

### 4.2. Country and institutions network analysis

Our analysis showed that the USA was the most collaborative and prolific country in this research area, and institutions like the University System of Ohio and the University of Texas System were identified as highly influential. The USA’s leading role can be attributed to its significant investment in healthcare research and nursing education, as well as its established infrastructure for research collaborations across academic and healthcare institutions.^[18]^ However, 58 of 78 countries and 298 of 302 institutions showed centrality values of <0.1, indicating weak cooperation between countries and institutions. There is a need for a more systematic and organized approach that considers institutional characteristics to foster stronger global collaborations. Nursing administrators in educational and healthcare settings must take an active role in promoting patient safety education for nursing students, leveraging research resources, training initiatives, and collaborative research to enhance educational and clinical outcomes.^[19]^

### 4.3. Journal and cited references analysis

Journal analysis revealed that Nurse Education Today, Nurse Education in Practice, and Clinical Simulation in Nursing are the leading journals publishing articles on patient safety education for nursing students, with Nurse Education Today being the most cited. Researchers may prioritize these journals for future publications and review the existing literature on patient safety education research in nursing.

Cited references network analysis showed that patient safety, nursing education, and adverse events were the most prevalent themes, with adverse events, acute care, and medical students being the most cited topics. This suggests that adverse events and acute care are central to patient safety education for nursing students. These references represent significant studies that have contributed to shaping the current understanding of this field.

### 4.4. Keyword and cluster analysis

Keyword network analysis indicated that “patient safety,” “education,” and “nursing education” were the most frequently occurring keywords from 2013 to 2023. Burst point analysis highlighted that adverse events, acute care, and medical students have been the most researched topics over the past decade. These keywords and emerging topics suggest that future researchers may explore less-explored areas to expand the literature on patient safety education.^[14]^ Cluster analysis revealed that medical education was the most significant cluster, with related keywords such as healthcare, attitude, and communication. Simulation training, another significant cluster, is integral to the education of healthcare professionals, offering immersive learning experiences. However, more research is needed to ensure the safety and efficacy of simulation training in nursing education.^[20]^ COVID-19 has had a significant impact on the delivery of nursing training in medical education and the implementation of teaching programs. The use of simulation practice offers an opportunity for experiential and immersive learning in a safe and supportive environment that can be an alternative to real-life scenarios.^[21]^ However, the safety of simulation training has yet to be confirmed by more research.

Standardized patients were the third significant cluster, with the first 3 keywords associated being knowledge and experience, and critical thinking. The clustering results indicate that patient safety education research for nursing students is a feasible way to emphasize clinical nursing practice. It is evident that the quality of performances delivered by the standardized patients employed for clinical skills education has a significant impact on the overall effectiveness of the training. Consequently, it is vital to ensure that standardized patients are provided with consistent feedback. It is of particular importance to ascertain the perspectives of students immediately following their encounters with standardized patients. In order to guarantee the quality of an educational program in which standardized patient encounters occur, it is imperative that the performance of the standardized patients is evaluated.^[22]^ While standardized patients are increasingly employed in medical education, previous studies have identified several gaps in the literature. These include the lack of safe application, the hiring of standardized patients, the use of higher-level outcomes, the use of policies and procedures, and the experiences of students, facilitators, and standardized patients.^[23]^

### 4.5. Strengths, limitations, and future directions

This study represents the first bibliometric analysis of patient safety education for nursing students, providing a comprehensive overview of emerging research trends, global shifts in focus, and pivotal topics in this field. The findings contribute to advancing research discussions in patient safety education. However, there are limitations to this study. First, only publications from the Web of Science database were included, which may not provide a complete view of the research landscape. Second, the study focused on original articles, which might have resulted in the omission of some significant topics. Lastly, the study only included articles published in English, limiting its generalizability.

Future research could benefit from incorporating multiple databases to obtain a broader view of the literature. Additionally, expanding the search to include alternative terms and articles in languages other than English would enrich the analysis and provide a more comprehensive understanding of global research trends in patient safety education for nursing students.

## 5. Conclusion

This bibliometric analysis provides a comprehensive overview of the most important developments and major trends in patient safety education for nurses between 2013 and 2023. The results demonstrate that the number of publications in the field continues to grow, with a clear dominance by the United States and Ohio University, which is also the country with the most prolific researchers and the institution that publishes the most papers. The analysis also revealed that the journal with the highest number of nursing research publications was *Nurse Education Today*, while the British Medical Journal was the most influential. Furthermore, the most frequently occurring keywords in the field were “patient safety,” “education,” and “nursing education.” Additionally, the 3 most frequent themes were “medical education,” “simulation training,” and “standardized patients.” The findings of this study will assist nursing students, medical educators, researchers, and academics in identifying prospective collaborative research partner teams, institutions, and even countries for collaborative research. Furthermore, the data will facilitate an understanding of the current state of patient safety education literature in nursing. The results of the study will inform the planning of future patient safety education research in nursing and will contribute to the advancement of patient safety education in nursing.

## Acknowledgments

We appreciate the work of editors and anonymous reviewers. We also thank the 2023 Shanghai Education Science Research Program (Grant No.C2023145).

## Author contributions

**Conceptualization:** Ying Wang.

**Data curation:** Xin-Ping Hu.

**Formal analysis:** Ying Wang.

**Project administration:** Hua Mei.

**Resources:** Yi-Wen Chen.

**Software:** Yi-Wen Chen.

**Supervision:** Hua Mei.

**Validation:** Xin-Ping Hu.

**Visualization:** Ying Wang.

**Writing – review & editing:** Ying Wang, Hua Mei.
